# Development of Ryanodine Receptor (RyR) Inhibitors for Skeletal Muscle and Heart Diseases

**DOI:** 10.14789/jmj.JMJ22-0045-R

**Published:** 2023-04-26

**Authors:** HIROYUKI MATSUKAWA, TAKASHI MURAYAMA

**Affiliations:** 1 Department of Pharmacology, Juntendo University School of Medicine, Tokyo, Japan; 1 Department of Pharmacology, Juntendo University School of Medicine, Tokyo, Japan

**Keywords:** ryanodine receptor, Ca^2+^ release channel, drug development, high-throughput screening

## Abstract

Ryanodine receptors (RyR) are intracellular calcium (Ca^2+^) release channels on the sarcoplasmic reticulum of skeletal and cardiac muscles that play a central role in excitation-contraction coupling. Genetic mutations or posttranslational modifications of RyR causes hyperactivation of the channel, leading to various skeletal muscle and heart diseases. Currently, no specific treatments exist for most RyR-associated diseases. Recently, high-throughput screening (HTS) assays have been developed to identify potential candidates for treating RyR-related muscle diseases. These assays have successfully identified several compounds as novel RyR inhibitors, which are effective in animal models. In this review, we will focus on recent progress in HTS assays and discuss future perspectives of these promising approaches.

## Introduction

The ryanodine receptor (RyR) is a calcium (Ca^2+^) release channel present in the endo/sarcoplasmic reticulum of various cells including skeletal muscle, heart, and brain. It forms a huge (>2 MDa) homotetrameric protein complex that comprised a large cytoplasmic structure with six transmembrane segments at the C-terminus forming a cation-channel domain^1, 2)^. Three major isoforms (RyR1-3) of RyR have been identified in mammals: RyR1 is mainly present in skeletal muscle, RyR2 in the heart, and RyR3 in various tissues at small amounts^3-5)^. RyR is activated by Ca^2+^ to release Ca^2+^ from the ER, referred to as Ca^2+^-induced Ca^2+^ release^6, 7)^. RyR1 also mediates depolarization-induced Ca^2+^ release, which is gated via physical interaction with a L-type voltage-dependent Ca^2+^ channel, specifically, the dihydropyridine receptor^8, 9)^.

Genetic mutations in RyR cause various skeletal muscle and heart diseases, including malignant hyperthermia (MH) and central core disease for RyR1,^10, 11)^ and catecholaminergic polymorphic ventricular tachycardia (CPVT) and arrhythmogenic right ventricular cardiomyopathy for RyR2^12, 13)^. The predominant underlying mechanism for these diseases is hyperactivation of the channel. Hyperactivation of RyR by posttranslational modifications may also be implicated in several diseases such as muscular dystrophy and heart failure^14, 15)^. Therefore, RyR inhibitors are therapeutic candidates for these diseases.

**Figure 1:**
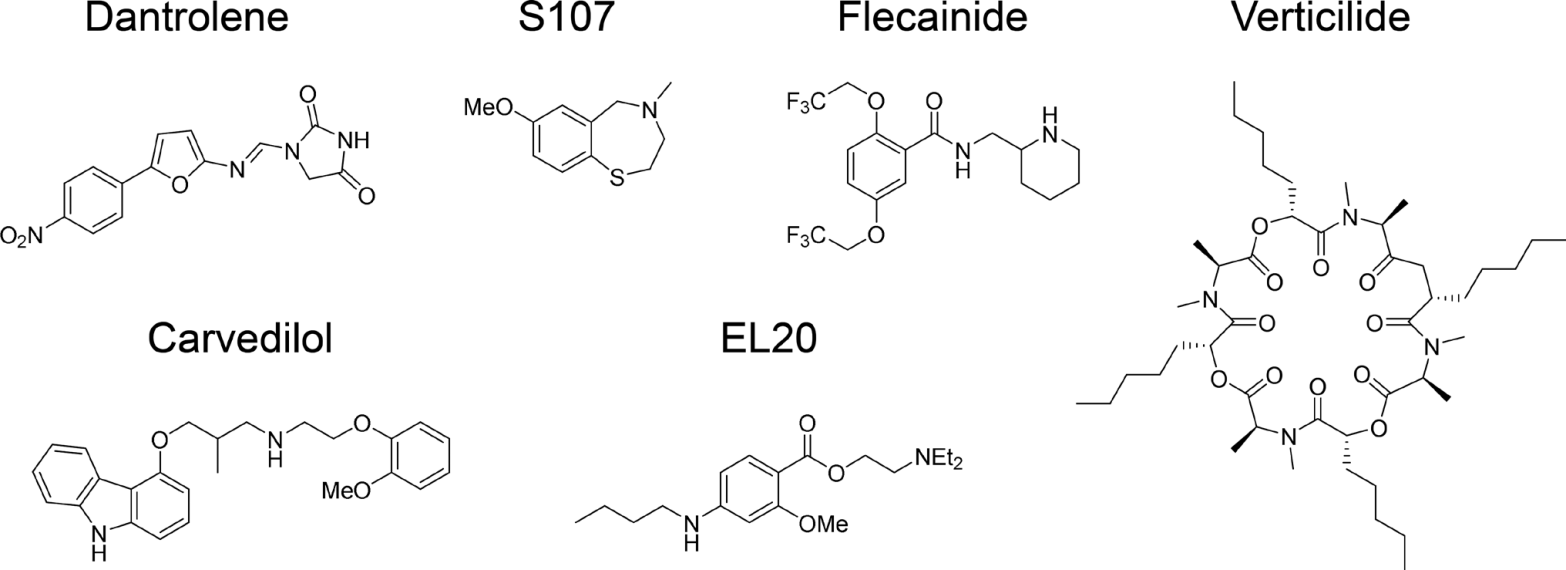
Structure of existing RyR inhibitors

In this review, we will briefly summarize several existing RyR inhibitors (Figure 1). Next, we will describe recent advances in high-throughput screening (HTS) for development of RyR inhibitors and discuss future perspectives.

## Existing RyR inhibitors

### Dantrolene

Dantrolene is the only approved drug for MH^16, 17)^. It was first synthesized in 1967 as a muscle relaxant^18)^. Later, it was found to prevent Ca^2+^ release by directly interacting with RyR1^19, 20)^. Dantrolene greatly reduced the mortality of patients with MH from 70-80% to < 10%^21)^. However, dantrolene has several disadvantages for clinical use including poor solubility in saline^22)^ and a long plasma half-life (~12 h), which prolongs side effects such as muscle weakness^23)^.

### Rycals

FK506 binding protein 12 (FKBP12) and FKBP12.6 bind to RyR1 and RyR2, respectively, to stabilize the channel, while posttranslational modifications (e.g., oxidation, nitrosylation, and phosphorylation) dissociate FKBP12/12.6 to cause leaky channels^15, 24)^. Rycals (S107 and S48168/ARM210) are benzothiazepine derivatives that prevent dissociation of FKBP12/12.6 from RyR channels^25, 26)^. Rycals exhibited therapeutic effects in various models of skeletal muscle diseases including RyR1-related myopathy, Duchenne muscular dystrophy, and sarcoglycanopathy, as well as in aging^27-29)^. A recent cryo-EM structure revealed the putative binding site of ARM210 in the RY1&2/P1 domain, to which it binds cooperatively with ATP to stabilize the closed state of RyR1^30)^.

### Flecainide

Flecainide is a class Ic antiarrhythmic drug that prolongs the duration of the cardiac action potential by blocking the sodium channel Nav1.5 in the heart^31)^. Watanabe and colleagues^32)^ reported that flecainide prevents mouse and human CPVT. Among class I antiarrhythmic agents, they found that only flecainide and propafenone showed antiarrhythmic activity against CPVT^33)^. They also showed that flecainide inhibited RyR2 channel activity^32, 33)^. Consistently, a N-methylated flecainide analog, which had less RyR2 inhibitory activity, did not prevent arrhythmias in CPVT mice^34)^. However, the effect of flecainide on RyR2 is still under debate^35)^. Bannister et al.^36)^ demonstrated that flecainide did not inhibit the physiologically relevant, luminal-to-cytosolic flux of cations through the RyR2 channel, although it partially blocked the cytosolic-to-luminal cation flux. Furthermore, Salvage et al.^37)^ recently demonstrated that flecainide activated RyR2 at lower concentrations, but was inhibitory at higher concentrations.

### Carvedilol

Carvedilol is a *β*-blocker used for chronic heart failure and CPVT. Compared with metoprolol, carvedilol extended the survival of patients with heart failure in clinical studies^38)^. Mochizuki et al.^39)^ reported that carvedilol improved intracellular Ca^2+^ concentration and systolic dysfunction in heart failure by correcting the interdomain interaction of RyR2. Zhou et al.^40)^ reported that carvedilol and its novel analogs (with minimal *β*-blocking activity) suppressed proarrhythmic store overload-induced Ca^2+^ release. This suggests that RyR2 inhibitory activity is responsible for the enhanced efficacy of carvedilol compared with other *β*-blockers in the treatment of arrhythmogenic heart diseases.

### EL20

Tetracaine is an ester-type local anesthetic and a nonselective RyR inhibitor within the millimolar range^41)^. Because tetracaine is a potent inhibitor for voltage-gated sodium (Na^+^) channels, it is not clinically used as a RyR inhibitor. Klipp et al.^42)^ synthesized derivatives of tetracaine and identified EL20, which suppressed RyR2 in the nanomolar range in the absence of calmodulin (CaM) in sheep. In R176Q CPVT model mice, EL20 suppressed the induction of ventricular tachycardia. Word et al.^43)^ further demonstrated that EL20 suppressed abnormal Ca^2+^ homeostasis in induced pluripotent stem cell- derived cardiomyocytes from a patient with CPVT. Since EL20 did not affect electrocardiogram parameters in wild-type mice, it may prevent CPVT without affecting conduction properties of the heart^42)^.

### Verticilide

Verticilide is a compound isolated from Verticillium sp. FKI-1033^44)^. Shiomi et al.^45)^ reported that verticilide inhibited RyR within the micromolar range, and more selectively inhibited insect RyR (half maximal inhibitory concentration [IC_50_] = 4.2 µM) than mammalian RyR2 (IC_50_ = 53.9 µM). Batiste et al.^46)^ synthesized derivatives of verticilide and found that an enantiomer (*ent*-1) inhibited RyR2 at the submicromolar range (IC_50_ = 0.1 µM). Furthermore, *ent*-1 significantly reduced spontaneous Ca^2+^ release in cardiomyocytes isolated from CPVT model mice and prevented ventricular arrhythmias, suggesting *ent*-1 may be a novel therapeutic candidate for CPVT. Structure-function relationships of *ent*-1 have shown that a high degree of N-methyl amide content is critical for its activity^47)^.

## High-throughput screening assays

As discussed, several RyR inhibitors have been identified and tested for the treatment of muscle and/or heart diseases. Most of these compounds also act on other targets (channels and receptors), which might cause side effects in clinical use. Therefore, the development of novel compounds that selectively inhibit RyR are an urgent need. High-throughput screening is a powerful method for the rapid evaluation of thousands to millions of chemical compounds. However, development of HTS assays targeted to RyR have been slowed by the lack of appropriate screening platforms. Recently, two groups developed HTS assays for RyR modulators using different approaches.

### Fluorescence energy transfer (FRET)-based HTS assay

RyR are tightly regulated by endogenous associated proteins, such as CaM and FKBP12/12.6^1, 2)^. Dissociation of these molecules from RyR may change its structural state, leading to Ca^2+^ leakage. Rebbeck et al.^48)^ developed a HTS assay for RyR1 modulators using time-resolved fluorescence resonance energy transfer (TR-FRET). They measured FRET between donor fluorospheres (bound to FKBP 12.6) and acceptor fluorospheres (bound to CaM). Substantial FRET signals were detected between the two proteins, reflecting their close proximity determined from structural data^49, 50)^. They screened a compound library consisting of 727 small molecule compounds and identified five compounds that significantly altered FRET. Of these, two compounds (tacrolimus and ebselen) were known RyR1 modulators, and three (cefatrizine PG, disulfiram, and chloroquine) were new RyR1 modulators^48)^. These hit compounds also showed RyR1 modulating activity in a [^3^H]ryanodine binding assay, suggesting that structure-based HTS assays are effective at detecting functional modulators of RyR1. Rebbeck et al.^51)^ further improved their strategy by miniaturizing the screening format to the industry standard of 1536-well plates. Using a larger library of 1,280 compounds, chloroxine and myricetin were identified as novel RyR1 inhibitors. They demonstrated that the two drugs significantly inhibited Ca^2+^ leakage from the sarcoplasmic reticulum via RyR1, with only slight effects on Ca^2+^ release in E-C coupling. Similar strategies between CaM and a biosensor peptide (DPc10) have been used to identify novel RyR2 inhibitors^52)^.

### Endoplasmic reticulum Ca^2+^-based HTS assay

**Figure 2:**
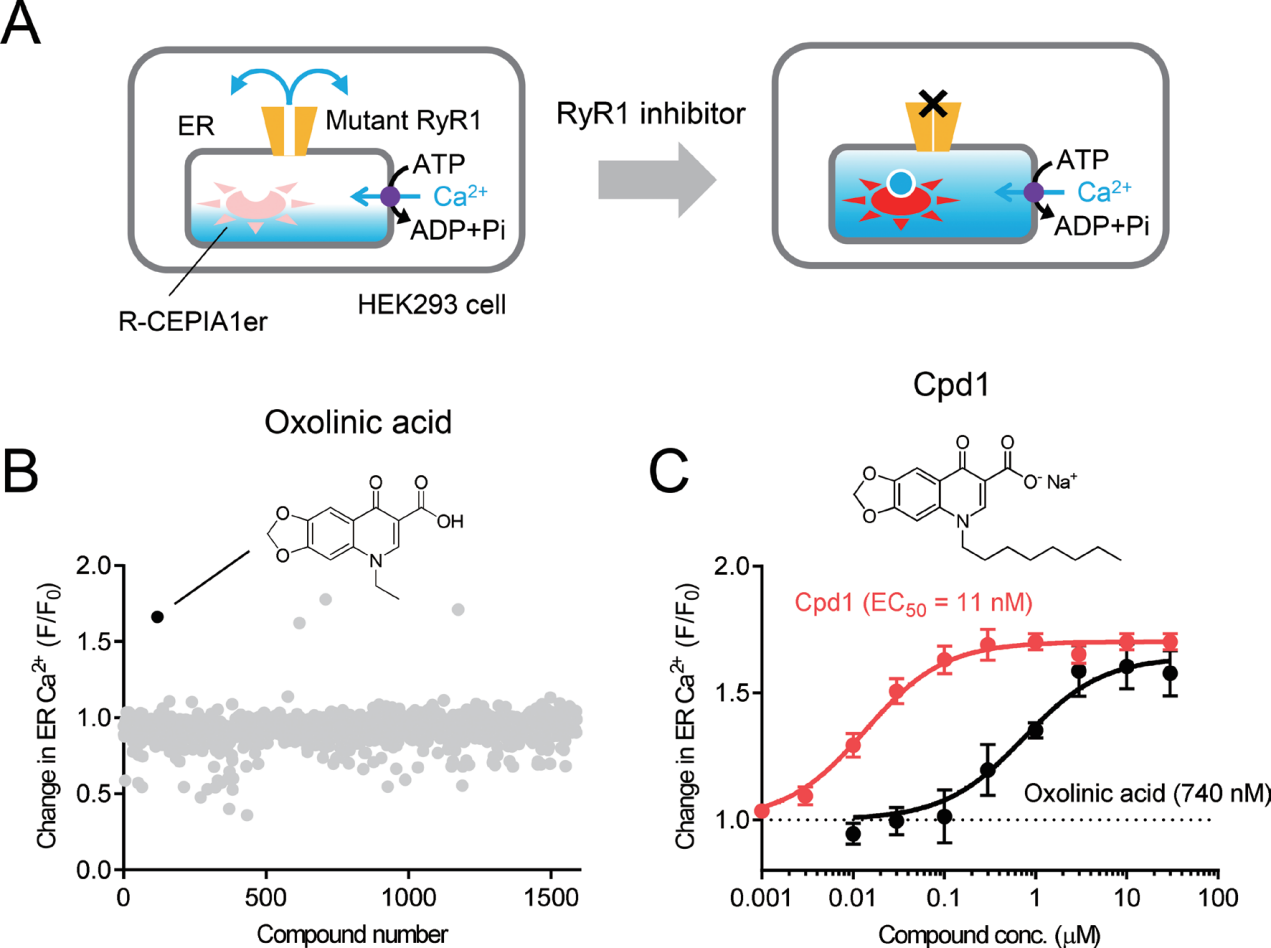
High-throughput assays for identifying RyR1 inhibitors by endoplasmic reticulum Ca^2+^ measurement (A) Schematic drawing of an endoplasmic reticulum (ER) Ca^2+^-based assay. Stable HEK293 cells were generated that express the gain-of-function mutant, RyR1, and R-CEPIA1er, a genetically encoded ER Ca^2+^ indicator. Ca^2+^ leakage via mutant RyR1 channels causes a reduction in ER Ca^2+^ content, therefore R-CEPIA1er fluorescence is low. RyR1 inhibitors prevent Ca^2+^ leakage, which causes an increase in ER Ca^2+^ content (via active transport through a Ca^2+^ pump) and increases R-CEPIA1er fluorescence. (B) Typical results for a highthroughput assay for RyR1 inhibitors. Oxolinic acid was identified and shown to increase R-CEPIA1er fluorescence. (C) Development of Cpd1. Among many oxolinic acid derivatives, Cpd1 exhibited 70-fold greater potency than oxolinic acid. (This figure and its legend were modified from Murayama et al., Mol Pharmacol, 2018; 94: 722-730^53)^; Murayama and Kurebayashi, Curr Protoc Pharmacol, 2019; 87: e71^54)^; Mori et al., Eur J Med Chem, 2019; 179: 837-848^59)^. The authors have permission to reproduce images from the copyright owner.)

Murayama and colleagues^53, 54)^ developed a HTS assay to search for novel RyR1 inhibitors using an endoplasmic reticulum (ER) Ca^2+^ concentration measurement ([Ca^2+^]_ER_) (Figure 2A). Generally, [Ca^2+^]_ER_ is determined by the balance between Ca^2+^ release by Ca^2+^ release channels (RyR or 1,4,5- trisphosphate [IP_3_] receptors) and Ca^2+^ uptake by sarco/endoplasmic reticulum Ca^2+^-ATPase (or SERCA) Ca^2+^ pumps. They found that under resting conditions, expression of hyperactive MH-mutant RyR1 reduces [Ca^2+^]_ER_ in HEK293 cells by Ca^2+^ leakage^55, 56)^. Addition of RyR1 inhibitors (dantrolene and tetracaine) to cells increased [Ca^2+^]_ER_ by preventing Ca^2+^ leakage^53)^. They measured [Ca^2+^]_ER_ using R-CEPIA1er, a fluorescent ER Ca^2+^ indicator^57)^, in a 96-well microplate reader. Using this assay platform, they screened a chemical compound library of well-characterized drugs (1,535 compounds) and identified several compounds as potential RyR1 inhibitors^53)^ (Figure 2B). Of these, oxolinic acid selectively inhibited RyR1 among the three RyR isoforms.

Oxolinic acid is a first-generation quinolone antibacterial drug that has been used to treat urinary tract infections with no major side effects^58)^. Mori et al.^59)^ synthesized a series of modifications to oxolinic acid at the 1-N position and benzene ring to successfully develop Cpd1, which exhibited > 70-fold greater potency (half maximal effective concentration [EC_50_] = 12 nM) than oxolinic acid (EC_50_ = 810 nM) (Figure 2C). Cpd1 preserved RyR1 selectivity among the three RyR isoforms. Ishida et al.^60)^ recently developed derivatives of oxolinic acid with greater water solubility.

**Figure 3:**
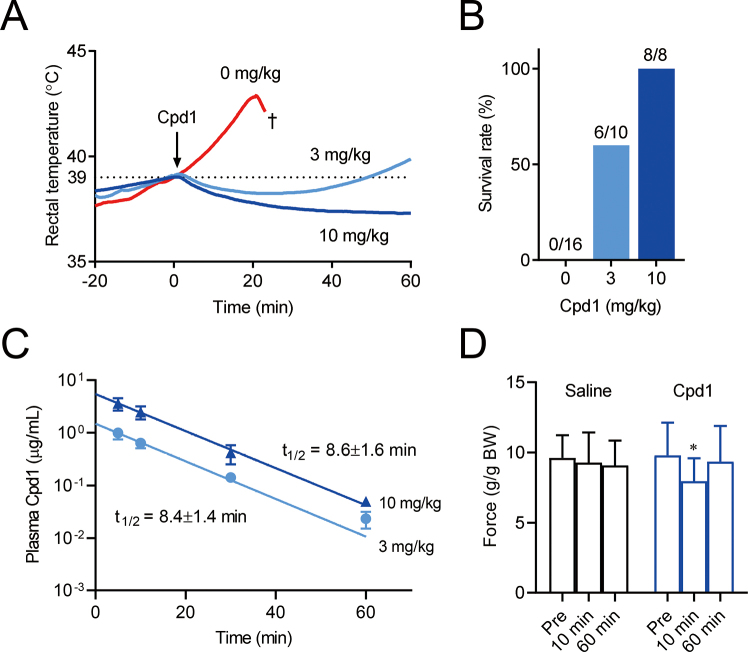
Therapeutic effects of Cpd1 on malignant hyperthermia model mice (A) Cpd1 was administered to malignant hyperthermia (MH) model mice during isofluraneinduced episodes of MH. Cpd1 reduced the body temperature of the mice. (B) Cpd1 improved survival rate in isoflurane-induced MH episodes. (C) Change in plasma Cpd1. Cpd1 rapidly declined from plasma with a half-life of ~10 min. (D) Cpd1 and muscle weakness in mice. Muscle weakness was observed at 10 min after administration of Cpd1, but disappeared by 60 min. (This figure and its legend were modified from Yamazawa T et al., Nat Commun, 2021; 12: 4293^61)^ under a CC BY license［ Creative Commons Attribution 4.0 International license］).

Yamazawa et al.^61)^ tested the therapeutic effects of Cpd1 using multiple MH mouse models carrying different RyR1 mutations (R163C, G2434R, and R2509C). Cpd1 effectively prevented and treated fulminant MH crisis and death triggered by isoflurane anesthesia (Figure 3A, B). It has been shown that environmental heat stress causes a rise in body temperature and death in MH model mice^62-65)^. Cpd1 effectively treated heat stroke and prevented death in MH mice^61)^. Low water solubility^22)^ and a long plasma half-life^23)^ are disadvantage of dantrolene in clinical use. Cpd1 showed > 30-fold greater solubility in saline (845 µg/mL) than dantrolene (26 µg/mL) and much faster clearance *in vivo* (t_1/2_ of ~10 min) compared with dantrolene (~10 h)^61)^ (Figure 3C, 3D). These findings suggest that Cpd1 has therapeutic effects *in vivo*, and certain advantages over dantrolene.

### Conclusions and future perspectives

Hyperactive RyR channels generated by genetic mutations or posttranslational modifications may cause various muscle and heart diseases. In addition to existing RyR inhibitors, novel RyR inhibitors are increasingly being identified by HTS approaches. Currently, two different methods are available, namely a FRET-based assay and ER Ca^2+^-based assay. Several compounds identified by these approaches are effective not only *in vitro* but also *in vivo* in animal models, indicating they are promising approaches.

HTS typically aims to screen hundreds of thousands to millions of compounds. Miniaturizing the screening format to 1536-well plates has been successful in a FRET-based assay^51)^ and is currently underway in an ER Ca^2+^-based assay. These improvements will accelerate identification of good hit compounds.

Recent advances in structural biology using cryo-EM have revealed the detailed structure of RyR channels including binding sites for ligands or drugs at near-atomic resolution^30, 66)^. Structure-based drug development could further improve drug affinity and selectivity, leading to the development of clinically useful, novel drugs in the near future.

RyR are also involved in the function of various tissues including brain, smooth muscles, and lymphocytes^3-5)^. Since RyR inhibitors might act on RyR in these tissues to cause side effects, caution must be taken in clinical use.

## Funding

This work was supported in part by Japan Society for the Promotion of Science KAKENHI (22H02805 to TM), an Intramural Research Grant for Neurological and Psychiatric Disorders from the National Center of Neurology and Psychiatry (2-5 to TM), and the Vehicle Racing Commemorative Foundation (6303 to TM).

## Author contributions

HM and TM wrote the manuscript. Both authors approved the final manuscript.

## Conflicts of interest statement

The authors declare that there are no conflicts of interest.
